# Lower invasiveness of papillary thyroid carcinoma associated with Hashimoto’s thyroiditis: evidence from AJCC 8th-edition stratification and a dose–response relationship with autoantibody titers

**DOI:** 10.3389/fendo.2026.1897756

**Published:** 2026-08-14

**Authors:** SiCong Zhou, Fei Gao, Wenbin Huang, Yiwen Ding, Bin Cao

**Affiliations:** 1 Department of Thyroid Vascular Surgery, Fujian Province Second People’s Hospital, The Second Affiliated Hospital of Fujian University of Traditional Chinese Medicine, Fuzhou, China; 2 Ultrasound Department, Fujian Province Second People’s Hospital, The Second Affiliated Hospital of Fujian University of Traditional Chinese Medicine, Fuzhou, China

**Keywords:** AJCC 8th edition, BRAF V600E, central lymph node metastasis, extrathyroidal extension, Hashimoto’s thyroiditis, papillary thyroid carcinoma, tumor immune microenvironment

## Abstract

**Background:**

Hashimoto’s thyroiditis (HT) and papillary thyroid carcinoma (PTC) frequently coexist, but the precise impact of HT on PTC invasiveness — particularly across the strict American Joint Committee on Cancer (AJCC) 8th-edition extrathyroidal extension (ETE) categories — remains incompletely characterized.

**Methods:**

We retrospectively analyzed 266 consecutive patients with histopathologically confirmed PTC (69 with concurrent HT; 197 without) who underwent thyroidectomy with routine prophylactic central neck dissection at our institution between March 2020 and December 2022. ETE was stratified per strict AJCC 8th-edition criteria into three tiers: No ETE (including pure thyroid capsule invasion), Microscopic ETE (perithyroidal fat invasion), and Gross ETE (T3b strap-muscle or T4 organ invasion). The primary multivariable models adjusted for age, sex, tumor diameter, and multifocality but deliberately excluded BRAF V600E mutation (treated as a mediator on the HT→aggressiveness pathway) to avoid overadjustment bias; secondary models including BRAF are also presented. Sensitivity analyses for nodal metrics included binary logistic regression for any CLNM and negative binomial regression for positive node count with log(nodal yield) offset.

**Results:**

Under strict AJCC 8 reclassification, gross ETE was observed in 10.7% of HT(−) versus 4.3% of HT(+) patients (adjusted OR = 0.21, 95% CI 0.06–0.81, P = 0.024). HT was an independent protective factor for ETE severity in ordinal regression (adjusted OR = 0.41, 95% CI 0.19–0.88, P = 0.023) and for BRAF V600E mutation (adjusted OR = 0.47, 95% CI 0.25–0.88, P = 0.018). High CLNM ratio (≥0.4) was less frequent in HT(+) (adjusted OR = 0.47, 95% CI 0.23–0.93, P = 0.029), and yield-adjusted negative binomial regression confirmed HT independently reduced the positive node count (IRR = 0.56, 95% CI 0.40–0.78, P < 0.001). The proportion with any CLNM did not differ between groups (62.9% vs. 55.1%, P = 0.313), nor did Ki-67 ≥5% (27.4% vs. 26.9%, P = 1.000). The proportional-odds assumption for ordinal regression was supported (Brant-type tests, all P > 0.30). Exploratory analyses across TPOAb strata suggested a stepwise gradient with BRAF V600E (Ptrend = 0.020) but were limited by very small subgroup sizes.

**Conclusions:**

In this Chinese single-center cohort, HT was independently associated with a less aggressive PTC phenotype — reduced gross ETE, lower BRAF V600E mutation, and reduced nodal metastatic burden — after applying strict AJCC 8 criteria and a causal-inference-informed analytic strategy. These observations strengthen the biological hypothesis that HT modulates PTC behavior, but require prospective multi-center validation with recurrence and survival endpoints before any clinical management changes.

## 1 Introduction

Thyroid cancer is the most common endocrine malignancy worldwide, with rising age-standardized incidence rates over the past two decades (1). Papillary thyroid carcinoma (PTC) is the most prevalent histological subtype, accounting for over 80% of all thyroid malignancies (2), and is characterized by indolent biology in most cases but with a clinically meaningful minority exhibiting aggressive features including extrathyroidal extension (ETE), regional lymph node metastasis, and tumor recurrence (2, 3).

Hashimoto’s thyroiditis (HT), the most common autoimmune thyroid disorder (4), frequently coexists with PTC — reportedly in approximately 10–40% of surgical PTC cohorts (5). Despite this well-established co-occurrence, the biological consequences of HT for PTC behavior remain debated (6, 7). A growing body of evidence, including recent large meta-analyses, suggests that HT-associated PTC (PTC-HT) exhibits a less aggressive phenotype, with reduced BRAF V600E mutation rates (8), lower lymph node metastasis (9–11), and improved disease-specific survival (7, 10). However, prior studies have varied substantially in their pathological definitions of ETE, their adjustment strategies for molecular drivers, and their handling of nodal-yield confounding.

While multiple large cohorts (Ma et al., 2025, n=6,963 (10); Janicki et al., 2023 meta-analysis, n=6,395 (8); Zhong et al., 2025 PTMC meta-analysis (11)) have established that HT-associated PTC exhibits less aggressive features, the present study contributes three methodological refinements that, to our knowledge, have not been combined in prior reports: (i) strict AJCC 8th-edition ETE stratification (12, 13) distinguishing thyroid capsule invasion (no ETE), perithyroidal fat invasion (microscopic ETE), and major adjacent organ invasion (gross ETE/T3b–T4), modeled as an ordinal outcome rather than dichotomized; (ii) a causal-inference-informed analytic strategy that excludes BRAF V600E from primary multivariable models (treating it as a mediator on the HT→aggressiveness pathway) to avoid overadjustment bias; and (iii) exploratory dose-response analyses across TPOAb strata. Although our sample size is more modest than the largest published cohorts, this methodological approach permits a refined biological interpretation of how HT may modulate PTC behavior.

In this study, we tested the hypothesis that HT is associated with reduced PTC invasiveness across multiple biological levels — molecular (BRAF V600E), local invasion (AJCC 8 ETE severity), regional metastasis (central lymph node burden), and proliferation (Ki-67 labeling index) — in a Chinese single-center cohort of 266 consecutive surgical PTC patients with uniform application of prophylactic central neck dissection.

## 2 Materials and methods

### 2.1 Study design and patient population

We conducted a retrospective single-center cohort study at The Second Affiliated Hospital of Fujian University of Traditional Chinese Medicine (Fuzhou, China). Consecutive adult patients (≥18 years) who underwent thyroidectomy for histologically confirmed PTC between March 2020 and December 2022 were screened. Exclusion criteria were: (i) non-PTC histology (follicular, medullary, or anaplastic thyroid carcinoma); (ii) previous thyroid surgery; (iii) concurrent non-thyroid malignancies; (iv) incomplete pathology data; and (v) insufficient clinical information. The final analytic cohort comprised 266 patients, of whom 69 (25.9%) had concurrent HT and 197 (74.1%) did not.

At our institution, prophylactic central neck dissection (pCND) was routinely performed for all patients undergoing thyroidectomy for histologically confirmed PTC during the study period. No patient was selected based on clinical or imaging suspicion of nodal disease; therapeutic neck dissection of lateral compartments was performed only when preoperative imaging demonstrated suspicious lateral nodal involvement.

The study protocol was approved by the institutional ethics committee (approval number SPHFJP-Y2025044-01) and was conducted in accordance with the Declaration of Helsinki. Given the retrospective nature, the requirement for written informed consent was waived.

### 2.2 Clinical and laboratory data collection

Demographic data (age, sex), preoperative thyroid function tests (TSH), and autoantibody titers (anti-thyroglobulin antibody [TgAb] and anti-thyroid peroxidase antibody [TPOAb]) were extracted from electronic medical records. Antibody positivity was defined using standard clinical cutoffs: TgAb > 115 IU/mL and TPOAb > 34 IU/mL.

### 2.3 Imaging and pathological assessment

Preoperative imaging variables comprised the American College of Radiology Thyroid Imaging Reporting and Data System (ACR TI-RADS), as defined by Tessler et al. (2017) (14), the maximum tumor diameter on ultrasonography, and a taller-than-wide shape on transverse view. The diagnosis of HT was based on postoperative histopathology: the non-neoplastic thyroid parenchyma showed diffuse lymphocytic infiltration with germinal-center formation and oncocytic (formerly Hürthle-cell) change, in accordance with the WHO 2022 Classification of Endocrine and Neuroendocrine Tumours (5th edition) (15).

Extrathyroidal extension (ETE) was strictly classified per the AJCC 8th edition (12, 13) into three tiers (Supplementary Figure S1):

- **No ETE**: cases with thyroid capsule invasion only (involvement of the thyroid’s own fibrous capsule without penetration beyond it), or no invasion. Per AJCC 8, capsule invasion alone does not constitute ETE.
- **Microscopic ETE**: cases with documented perithyroidal fat invasion (extrathyroidal soft tissue). Microscopic ETE per AJCC 8 is no longer used for T-stage upgrading.
- **Gross ETE (T3b/T4)**: cases with strap muscle invasion (T3b) or invasion of larger adjacent organs (larynx, trachea, esophagus, recurrent laryngeal nerve, etc.; T4).

Pathology slides were independently re-reviewed by two senior endocrine pathologists for ETE classification and central lymph node status. No case required re-classification compared with the original pathology reports; inter-rater agreement was 100% concordant for both ETE tier assignment and nodal status. Multifocality was defined as ≥2 distinct PTC foci; bilaterality as foci in both thyroid lobes. Central lymph node metastasis (CLNM) was characterized by the number of harvested nodes, number of metastatic nodes, and CLNM ratio (positive/total); a CLNM ratio ≥0.4 was prespecified as ‘high.’

BRAF V600E mutation was determined preoperatively from fine-needle aspiration biopsy material by amplification-refractory mutation system PCR. Ki-67 immunohistochemistry was performed on 4-µm formalin-fixed paraffin-embedded sections using a rabbit monoclonal anti-Ki-67 antibody (clone SP6; GeneTex, Shanghai, China) according to the manufacturer’s recommended protocol. The Ki-67 labeling index was independently assessed by two pathologists by counting positively stained tumor nuclei in the areas showing the highest proliferative activity (‘hot spot’ method), with at least 500 tumor cells evaluated per case at ×400 magnification. Per institutional clinical reporting convention, Ki-67 values below 5% were reported as ‘<5%’ (rather than as exact percentages), and Ki-67 ≥5% was defined as ‘high’ for binary analyses.

### 2.4 Statistical analysis

Continuous variables were presented as median (Q1, Q3) and compared by the Mann–Whitney U test; categorical variables were presented as n (%) and compared by the chi-square test, or Fisher’s exact test when any expected count was <5.

Variable selection for multivariable analysis followed a causal-inference framework. Our primary multivariable models for tumor-aggressiveness outcomes (ETE severity, gross ETE, CLNM metrics) adjusted for age, sex, tumor maximum diameter, and multifocality, but deliberately did not include BRAF V600E mutation status as a covariate. This decision reflects the directed acyclic graph (DAG) (Supplementary Figure S2) depicting HT, BRAF, and tumor aggressiveness in PTC: BRAF V600E is hypothesized to lie on the causal pathway between HT and reduced tumor aggressiveness (HT → reduced BRAF → reduced aggressiveness) (16, 17), and adjusting for an intermediate variable would introduce overadjustment bias and attenuate the estimated total causal effect of HT. For transparency, secondary models including BRAF as a covariate are also presented in **Table 1**.

**Table 1:** Multivariable regression models for the association of Hashimoto’s thyroiditis with key invasive features (AJCC 8 strict classification).

Variable	Primary model (without BRAF) ^a^	Secondary model (with BRAF)	Sig.
Model 1 — Ordinal logistic: ETE severity tier (0/1/2); n = 266			
Hashimoto’s thyroiditis	0.408 (0.188–0.884) P = 0.023	0.490 (0.225–1.067) P = 0.073	*
Age, per year	0.984 (0.962–1.007) P = 0.169	0.982 (0.960–1.005) P = 0.132	
Male sex	0.430 (0.204–0.904) P = 0.026	0.421 (0.199–0.889) P = 0.023	*
Pathological max diameter, per mm	1.056 (1.022–1.092) P = 0.001	1.058 (1.023–1.095) P = 0.001	***
Multifocality	0.931 (0.483–1.795) P = 0.832	0.840 (0.432–1.633) P = 0.607	
BRAF V600E	—	2.786 (1.189–6.527) P = 0.018	*
Model 2 — Binary logistic: Gross ETE (T3b/T4); n = 266			
Hashimoto’s thyroiditis	0.211 (0.055–0.813) P = 0.024	0.225 (0.056–0.899) P = 0.035	*
Age, per year	0.983 (0.951–1.015) P = 0.291	0.982 (0.951–1.015) P = 0.282	
Male sex	0.213 (0.056–0.804) P = 0.023	0.213 (0.056–0.805) P = 0.023	*
Pathological max diameter, per mm	1.071 (1.025–1.119) P = 0.002	1.070 (1.024–1.118) P = 0.002	**
Multifocality	0.769 (0.281–2.108) P = 0.610	0.754 (0.273–2.079) P = 0.585	
BRAF V600E	—	1.210 (0.399–3.669) P = 0.736	
Model 3 — Binary logistic: High CLNM ratio (≥0.4); n = 261 ^b^			
Hashimoto’s thyroiditis	0.465 (0.234–0.925) P = 0.029	—	*
Age, per year	0.957 (0.936–0.979) P = <0.001	—	***
Male sex	2.091 (1.120–3.906) P = 0.021	—	*
Pathological max diameter, per mm	1.045 (1.009–1.082) P = 0.015	—	*
Multifocality	0.754 (0.407–1.397) P = 0.370	—	
Gross ETE (T3b/T4)	4.070 (1.513–10.952) P = 0.005	—	**
Model 4 — Binary logistic: BRAF V600E mutation; n = 266			
Hashimoto’s thyroiditis	0.473 (0.254–0.879) P = 0.018	—	*
Age, per year	1.007 (0.985–1.030) P = 0.520	—	
Male sex	1.257 (0.635–2.487) P = 0.512	—	
Pathological max diameter, per mm	1.001 (0.967–1.036) P = 0.954	—	
Multifocality	2.005 (1.010–3.981) P = 0.047	—	*
Model 5 — Negative binomial: Positive node count (offset for log[nodal yield]); n = 261 ^c^			
Hashimoto’s thyroiditis	0.559 (0.402–0.776) P = <0.001	—	***
Age, per year	0.978 (0.968–0.989) P = <0.001	—	***
Male sex	1.332 (0.972–1.825) P = 0.075	—	
Pathological max diameter, per mm	1.020 (1.005–1.036) P = 0.010	—	*
Multifocality	1.038 (0.768–1.402) P = 0.810	—	
Gross ETE (T3b/T4)	1.411 (0.915–2.177) P = 0.119	—	

All effect estimates are adjusted odds ratios (OR) for Models 1–4 or incidence rate ratios (IRR) for Model 5, with 95% confidence intervals in parentheses. ^a^ Primary models follow a causal-inference framework, excluding BRAF V600E because it is hypothesized to lie on the causal pathway HT → reduced BRAF → reduced tumor aggressiveness, where adjusting for a mediator introduces overadjustment bias. Secondary models include BRAF and are shown for transparency only. ^b^ Model 3 additionally adjusts for gross ETE because nodal burden is biologically downstream of ETE extent. ^c^ Model 5 (negative binomial regression with log(nodal yield) as offset) addresses the concern that higher CLNM ratio in HT(−) might partly reflect their lower nodal yield. Proportional odds assumption for Model 1 supported by Brant-type tests (all P > 0.30). *P < 0.05; **P < 0.01; ***P < 0.001.

Primary models exclude BRAF V600E to avoid overadjustment bias (BRAF as mediator); secondary models with BRAF are shown for transparency.

ETE severity (3-tier) was modeled by proportional-odds ordinal logistic regression. The proportional-odds assumption was assessed via Brant-type tests (Supplementary Table S1) comparing coefficients across cumulative cutpoints; non-significant tests (all P > 0.30) supported the assumption. Binary outcomes (gross ETE, BRAF V600E mutation, high CLNM ratio) were modeled by logistic regression. To address the concern that higher CLNM ratio in HT(−) patients might partly reflect their lower nodal yield rather than greater metastatic burden, we additionally performed: (i) binary logistic regression for the presence of any CLNM; and (ii) negative binomial regression for the positive node count with log(nodal total) included as offset, providing a yield-adjusted measure of metastatic burden.

Exploratory dose-response patterns across TPOAb strata (negative ≤34 IU/mL; weakly positive 35–100 IU/mL; positive >100 IU/mL) were assessed using the Cochran-Armitage trend test and Spearman correlations with log-transformed antibody titers. All analyses were two-sided, with statistical significance set at P < 0.05. Analyses were performed in Python (version 3.12) using pandas, scipy, and statsmodels.

**AI usage disclosure:** Statistical code development and figure-plotting code were generated with the assistance of large-language-model tools (Claude, Anthropic). The authors used Claude for English language editing and PubMed reference verification. All AI-assisted outputs were manually verified by the authors, who assume full responsibility for the integrity and accuracy of the data, analyses, and conclusions. No AI tools were used to generate primary research data, synthetic images, or pathological illustrations.

## 3 Results

### 3.1 Baseline characteristics

Among 266 consecutive PTC patients, 69 (25.9%) had concurrent HT and 197 (74.1%) did not. Baseline clinical and pathological characteristics are summarized in **Table 2**. HT(+) patients were younger (median 43 vs. 47 years, P = 0.020) and predominantly female (8.7% male vs. 34.0%, P < 0.001). Preoperative TSH was higher in HT(+) (median 2.01 vs. 1.42 mIU/L, P = 0.001), as expected. TgAb and TPOAb titers and positivity rates were dramatically elevated in HT(+) (P < 0.001 for all). ACR TI-RADS category, ultrasonographic maximum diameter, pathological maximum diameter, the proportion with tumors ≥10 mm, multifocality, and bilaterality did not differ significantly between groups.

**Table 2:** Baseline clinical and pathological characteristics of patients with PTC, stratified by Hashimoto’s thyroiditis (HT) status.

Variable	HT(−) (n = 197)	HT(+) (n = 69)	Test	P value
Demographics				
Age, years	47 (37, 57)	43 (33, 51)	MW	0.020*
Male sex, n (%)	67 (34.0)	6 (8.7)	χ²	<0.001***
Thyroid function and autoantibodies				
TSH, mIU/L	1.42 (0.98, 1.94)	2.01 (1.21, 2.62)	MW	0.001***
TgAb, IU/mL	0.10 (0.10, 0.10)	13.60 (1.90, 65.90)	MW	<0.001***
TPOAb, IU/mL	0.70 (0.40, 1.20)	23.00 (3.90, 263.30)	MW	<0.001***
TgAb positive (>115 IU/mL), n (%)	3 (1.5)	15 (21.7)	Fisher	<0.001***
TPOAb positive (>34 IU/mL), n (%)	3 (1.5)	31 (44.9)	Fisher	<0.001***
Preoperative imaging				
ACR TI-RADS category	5 (5, 5)	5 (5, 5)	MW	0.124
US maximum diameter, mm ^a^	8.80 (6.10, 14.72)	11.25 (6.98, 15.00)	MW	0.140
Molecular marker				
BRAF V600E mutation, n (%)	154 (78.2)	42 (60.9)	χ²	0.008**
Tumor size and distribution				
Pathological max diameter, mm	9.20 (6.50, 15.60)	10.60 (7.20, 13.60)	MW	0.566
Tumor ≥10 mm, n (%)	91 (46.2)	39 (56.5)	χ²	0.181
Multifocality, n (%)	57 (28.9)	18 (26.1)	χ²	0.767
Bilaterality, n (%)	40 (20.3)	18 (26.1)	χ²	0.406
Invasiveness (AJCC 8 strict) ^b^				
Any ETE, n (%)	48 (24.4)	11 (15.9)	χ²	0.200
Microscopic ETE (perithyroidal fat), n (%)	27 (13.7)	8 (11.6)	χ²	0.811
Gross ETE (T3b/T4), n (%)	21 (10.7)	3 (4.3)	Fisher	0.145
Central lymph node metastasis ^c^				
Total LNs dissected	6 (3, 9)	8 (5, 13)	MW	<0.001***
Positive LNs (count)	1 (0, 4)	1 (0, 3)	MW	0.231
Any CLNM, n (%)	124 (62.9)	38 (55.1)	χ²	0.313
CLNM ratio	0.28 (0.00, 0.65)	0.11 (0.00, 0.40)	MW	0.006**
High CLNM ratio (≥0.4), n (%) ^d^	82 (42.7)	18 (26.1)	χ²	0.022*
Proliferative activity ^e^				
Ki-67 index, % ^f^	4.5 (4.5, 5.0)	4.5 (4.0, 5.0)	MW	0.654
High Ki-67 (≥5%), n (%)	52 (27.4)	18 (26.9)	χ²	1.000

Continuous variables are presented as median (Q1, Q3) and compared by the Mann–Whitney U (MW) test; categorical variables as n (%) and compared by the χ² test, or Fisher’s exact test when any expected count was <5. ^a^ US maximum diameter missing for some patients (effective n = 156/50). ^b^ Invasiveness was reclassified per the strict AJCC 8th edition: pure thyroid capsule invasion (without extension beyond the capsule) does not constitute ETE; microscopic ETE = perithyroidal fat invasion (no T-stage upgrading); gross ETE = strap muscle (T3b) or larger adjacent organ invasion (T4). ^c^ All 266 patients underwent routine prophylactic central neck dissection at our institution during the study period. ^d^ CLNM ratio missing for 5 patients (effective n = 192/69). ^e^ Ki-67 missing for 7 HT(−) and 2 HT(+) patients (effective n = 190/67). ^f^ Ki-67 values reported as “<5%” in pathology reports (n = 137; institutional convention indicating low proliferation) were assigned a value of 4.5% for the continuous summary; binary categorization as Ki-67 ≥5% was based on whether the original report exceeded the 5% threshold explicitly. *P < 0.05; **P < 0.01; ***P < 0.001.

ETE classification follows strict AJCC 8th-edition criteria.

**Figure 1:**
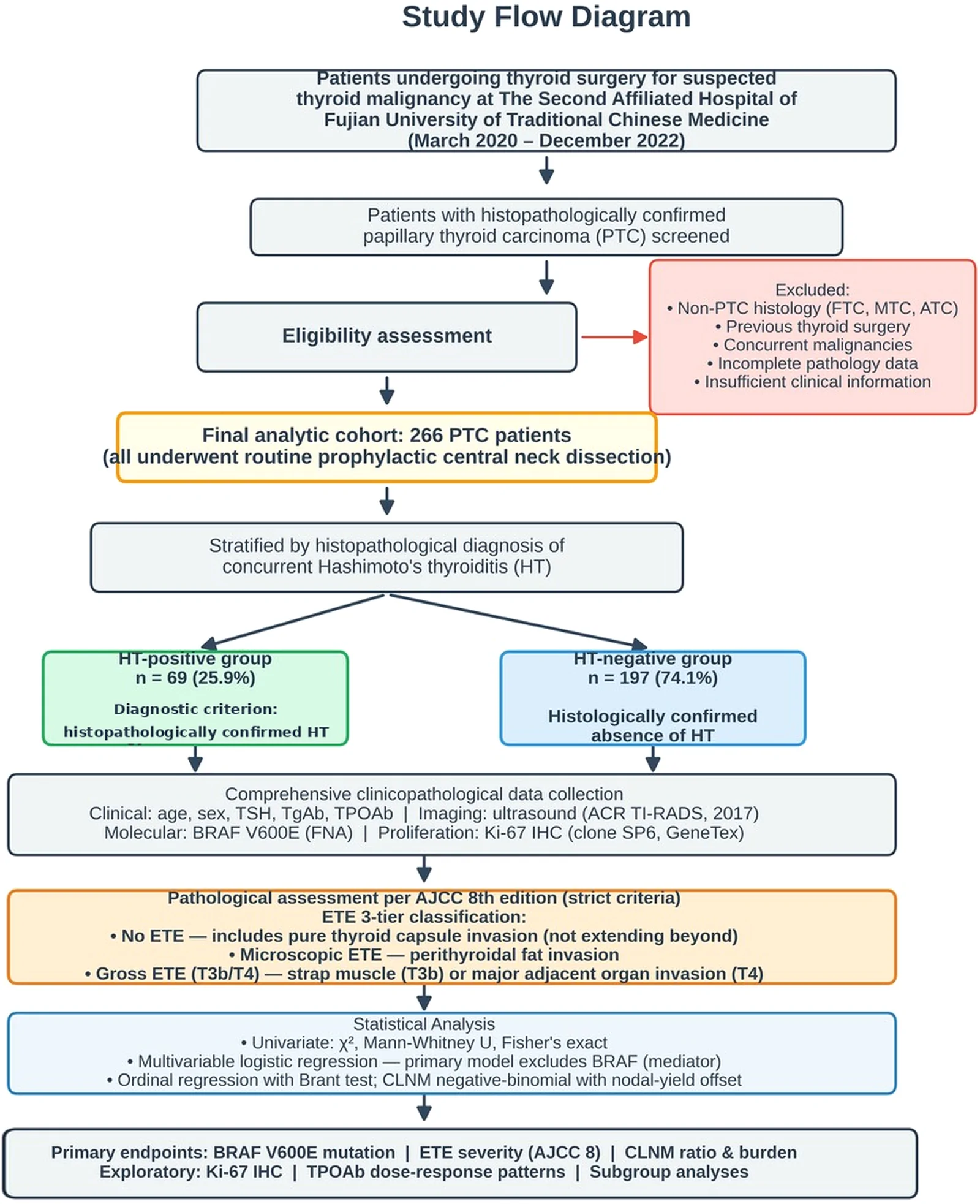
Study flow diagram. CONSORT-style schematic summarizing patient identification, eligibility, exclusion criteria, stratification by Hashimoto’s thyroiditis (HT) status, comprehensive clinicopathological data collection, AJCC 8th-edition strict pathological assessment, and statistical analysis plan. The final analytic cohort comprised 266 patients with histopathologically confirmed PTC, of whom 69 (25.9%) had concurrent HT and 197 (74.1%) did not. All 266 patients underwent routine prophylactic central neck dissection at our institution during the study period.

The flow of patient identification, eligibility, and stratification is depicted in **Figure 1**.

### 3.2 BRAF V600E mutation

BRAF V600E mutation was detected in 154/197 HT(−) patients (78.2%) and 42/69 HT(+) patients (60.9%) — a significant unadjusted difference (χ² P = 0.008). In multivariable logistic regression adjusted for age, sex, pathological diameter, and multifocality, HT remained an independent protective factor against BRAF V600E mutation (adjusted OR = 0.47, 95% CI 0.25–0.88, P = 0.018; **Table 1**, Model 4).

### 3.3 Extrathyroidal extension under AJCC 8 strict criteria

**Figure 2:**
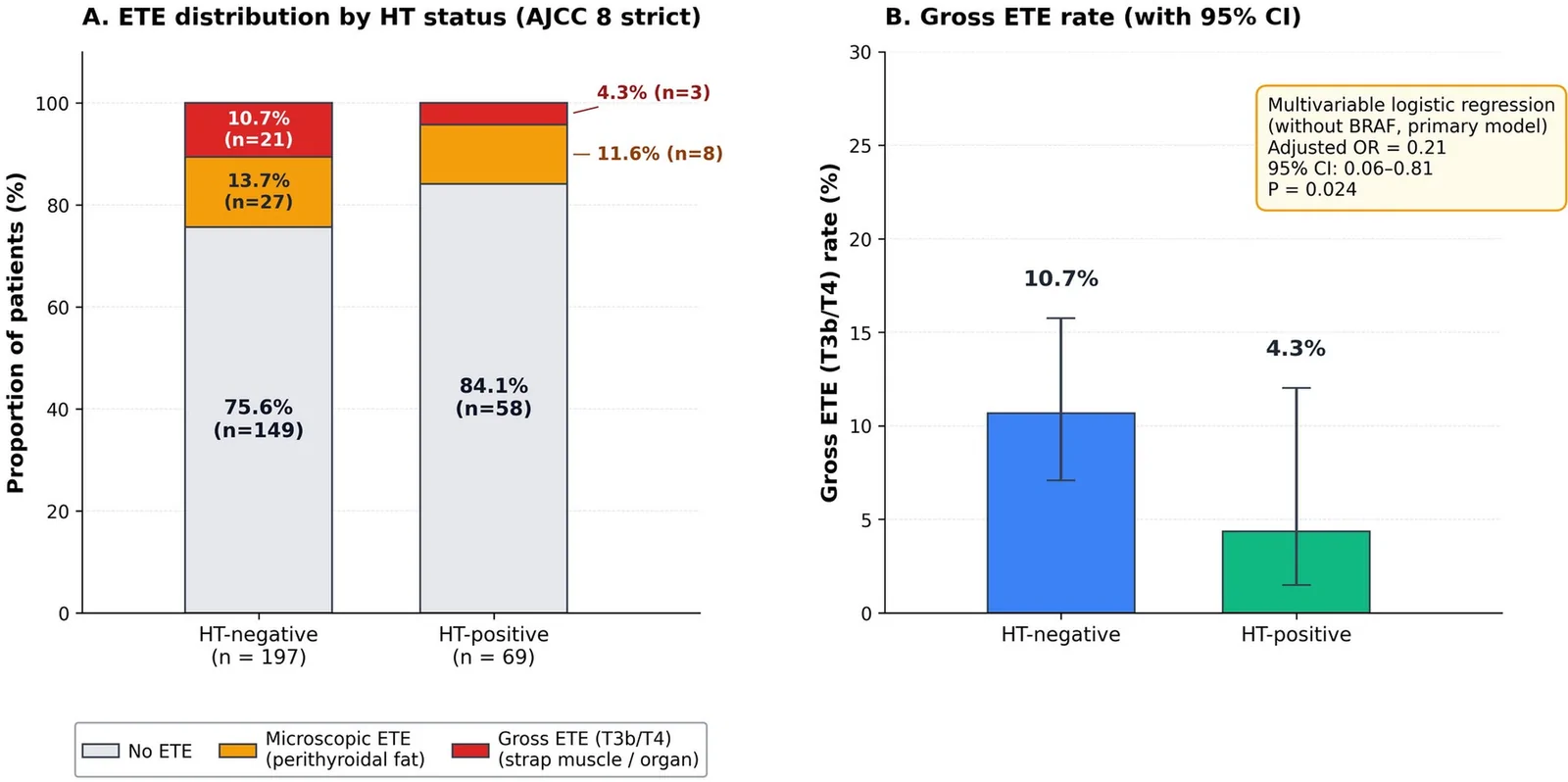
ETE distribution by HT status under strict AJCC 8th-edition criteria. **(A)** Stacked bar chart of the distribution of ETE severity tiers within each HT group: No ETE (light gray, includes pure capsule invasion), Microscopic ETE (orange, perithyroidal fat invasion), and Gross ETE (red, T3b/T4 — strap muscle or larger adjacent organ invasion). HT(−): 75.6%/13.7%/10.7% (n = 149/27/21). HT(+): 84.1%/11.6%/4.3% (n = 58/8/3). **(B)** Gross ETE (T3b/T4) rate with 95% Wilson confidence intervals: 10.7% (HT(−)) vs. 4.3% (HT(+)). Multivariable logistic regression — primary model (without BRAF adjustment, as BRAF is hypothesized to be a mediator) — yielded adjusted OR = 0.21 (95% CI 0.06–0.81, P = 0.024).

Under strict AJCC 8 reclassification, the distribution of ETE severity tiers differed between groups in the expected protective direction (**Figure 2A**). Among HT(−) patients: 149 (75.6%) had no ETE, 27 (13.7%) had microscopic ETE (perithyroidal fat invasion), and 21 (10.7%) had gross ETE (T3b/T4); the corresponding figures for HT(+) patients were 58 (84.1%), 8 (11.6%), and 3 (4.3%). The gross ETE rate was thus 2.5-fold higher in HT(−) than in HT(+) (**Figure 2B**).

In multivariable ordinal logistic regression (primary model, without BRAF), HT was an independent protective factor for ETE severity (adjusted OR = 0.41, 95% CI 0.19–0.88, P = 0.023; **Table 1**, Model 1). The proportional-odds assumption was supported by Brant-type tests (all covariates P > 0.30). Binary logistic regression for gross ETE specifically also showed HT as an independent protective factor (adjusted OR = 0.21, 95% CI 0.06–0.81, P = 0.024; **Table 1**, Model 2; **Figure 2B**). The secondary models additionally adjusted for BRAF V600E yielded attenuated effect estimates (ordinal: adjusted OR = 0.49, P = 0.073; gross ETE: adjusted OR = 0.23, P = 0.035), consistent with BRAF lying on the causal pathway.

### 3.4 Central lymph node metastasis

HT(+) patients had a greater number of harvested central lymph nodes (median 8 vs. 6, P < 0.001), reflecting the well-documented reactive lymphoid hyperplasia characteristic of autoimmune thyroiditis rather than a more aggressive surgical approach (5, 9). Notably, the proportion of patients with any CLNM did not differ significantly between groups (55.1% vs. 62.9%, P = 0.313), confirming that elevated harvest reflects nodal hyperplasia rather than greater metastatic burden. However, the CLNM ratio (positive/total) was lower in HT(+) (median 0.11 vs. 0.28, P = 0.006), and the proportion of patients with a high CLNM ratio (≥0.4) was significantly lower in HT(+) (26.1% vs. 42.7%, P = 0.022). In multivariable logistic regression adjusted for demographics, tumor diameter, multifocality, and gross ETE, HT remained an independent protective factor for high CLNM ratio (adjusted OR = 0.47, 95% CI 0.23–0.93, P = 0.029; **Table 1**, Model 3).

### 3.5 CLNM sensitivity analyses

To address the potential confounding effect of nodal yield on the CLNM ratio finding, we performed three complementary analyses (a–c) summarized in **Table 1**:

- (a) The proportion of patients with any CLNM did not differ significantly between groups: HT(−) 62.9% vs. HT(+) 55.1% (adjusted OR = 0.72, 95% CI 0.39–1.31, P = 0.278) — consistent with the hypothesis that the ratio effect could partly reflect nodal yield.
- (b) The high CLNM ratio (≥0.4) finding held in multivariable analysis (adjusted OR = 0.47, 95% CI 0.23–0.93, P = 0.029).
- (c) In a yield-adjusted analysis using negative binomial regression with log(nodal yield) as offset, HT independently reduced the positive node count (IRR = 0.56, 95% CI 0.40–0.78, P < 0.001; **Table 1**, Model 5), demonstrating that the protective effect is not an artefact of differing nodal retrieval.

Together, these analyses indicate that HT does not reduce the binary presence of CLNM but does reduce the depth/burden of nodal involvement when adjusted for nodal yield.

### 3.6 Exploratory dose-response patterns across TPOAb strata

**Figure 3:**
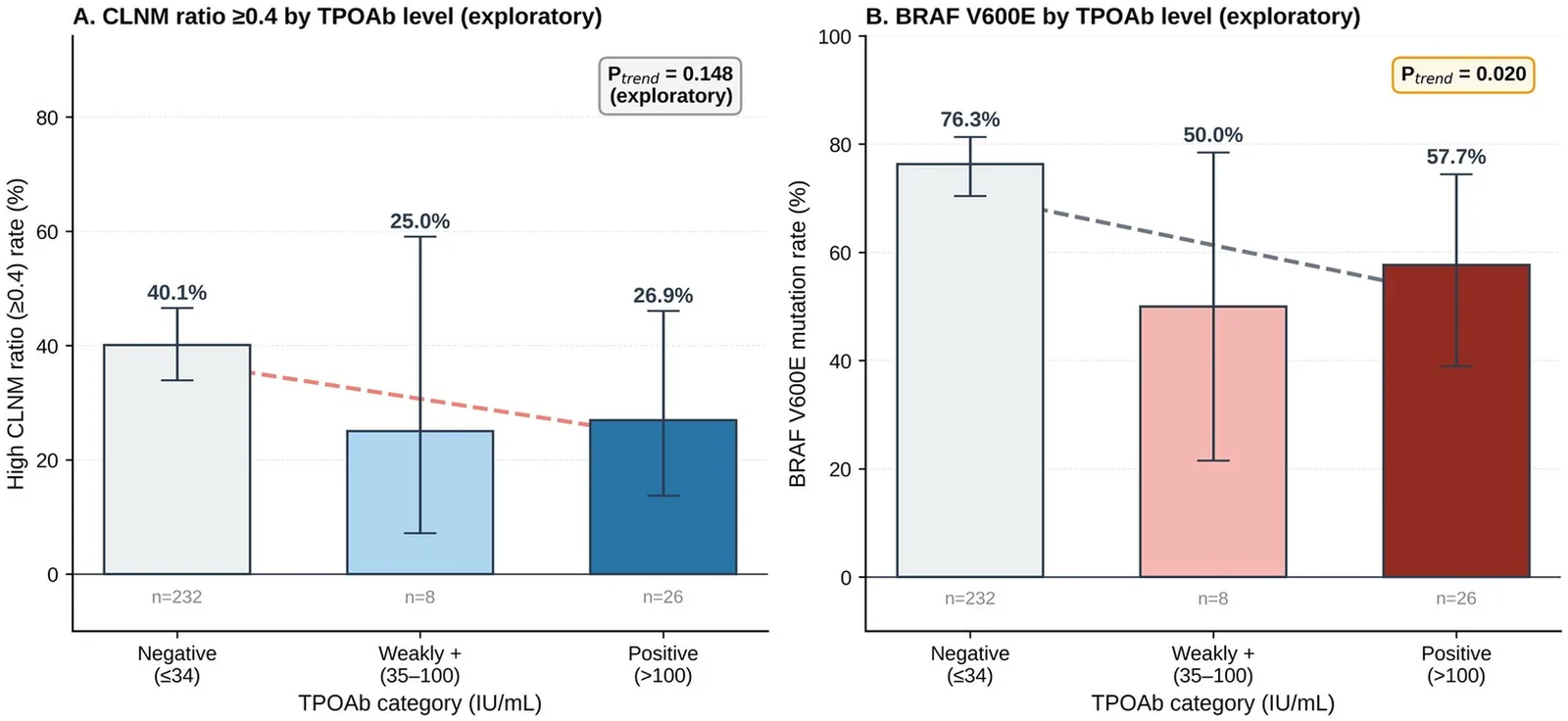
Exploratory dose-response patterns across TPOAb strata. Patients were grouped into three TPOAb categories: Negative (≤34 IU/mL, n = 232), Weakly positive (35–100 IU/mL, n = 8), and Positive (>100 IU/mL, n = 26). **(A)** High CLNM ratio (≥0.4) rate across strata: 40.1%, 25.0%, 26.9% (Ptrend = 0.148; exploratory). **(B)** BRAF V600E mutation rate across strata: 76.3%, 50.0%, 57.7% (Ptrend = 0.020). Error bars represent 95% Wilson confidence intervals; dashed lines indicate the linear trend. These analyses are explicitly exploratory and hypothesis-generating given the very small subgroup sizes.

As an exploratory, hypothesis-generating analysis, we examined whether tumor characteristics varied across TPOAb strata (**Figure 3**). The very small subgroup sizes (especially the intermediate category: weakly positive n = 8) limit statistical power; results should be interpreted as suggesting hypotheses for validation in larger cohorts rather than as evidence of a confirmed dose-response relationship. Patients were grouped into three categories: negative (≤34 IU/mL, n = 232), weakly positive (35–100 IU/mL, n = 8), and positive (>100 IU/mL, n = 26).

Across these three categories, the BRAF V600E mutation rate declined: 76.3%, 50.0%, and 57.7% (Cochran-Armitage trend P = 0.020; **Figure 3B**). The high-CLNM-ratio rate also declined: 40.1%, 25.0%, and 26.9% (trend P = 0.148; **Figure 3A**). Spearman correlations between log-transformed TPOAb (or log-TgAb) and outcomes were modest (r between −0.10 and −0.17 for the statistically significant pairs; e.g., log-TgAb × BRAF V600E: r = −0.17, P = 0.005). These findings should be interpreted cautiously given the small subgroup sizes.

### 3.7 Exploratory subgroup analyses

In an exploratory subgroup analysis restricted to tumors ≥10 mm (n = 130; HT(−) n = 91, HT(+) n = 39), the gross ETE rate was 11.0% in HT(−) versus 7.7% in HT(+), with no statistical evidence of effect modification by tumor size (HT × Tumor_ge10mm interaction P = 0.99). These subgroup findings should be interpreted as exploratory given the limited number of events (n = 13 gross ETE in this subgroup).

In multivariable analysis, HT was not associated with multifocality after adjustment (adjusted OR = 0.87, 95% CI 0.46–1.68, P = 0.69) but showed a borderline positive association with bilaterality (adjusted OR = 2.45, 95% CI 0.96–6.22, P = 0.060); multifocality was the dominant predictor of bilaterality (adjusted OR = 31.7, 95% CI 13.8–72.7, P < 0.001).

### 3.8 Summary forest plot

**Figure 4:**
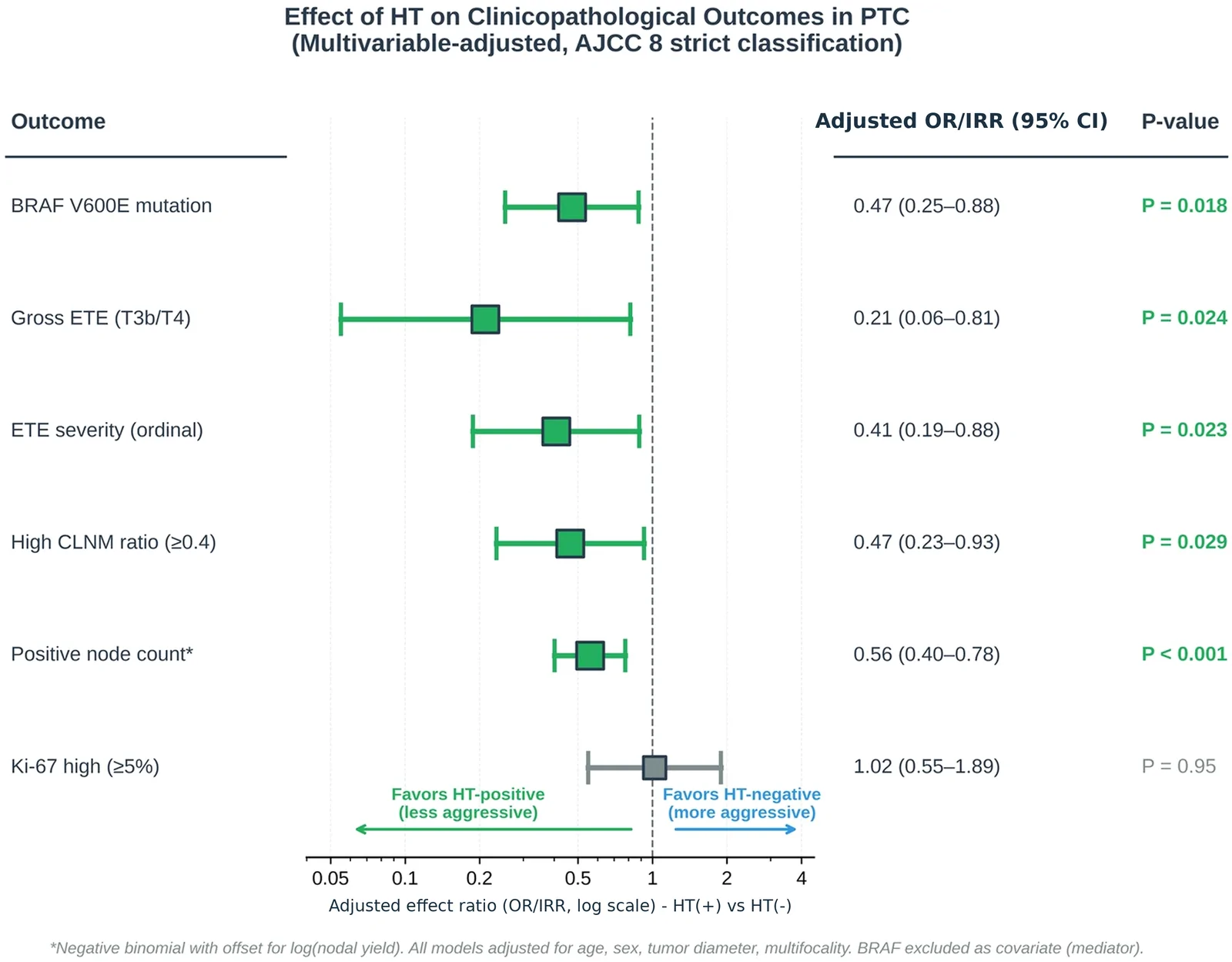
Multivariable-adjusted associations between Hashimoto’s thyroiditis and PTC invasive features (AJCC 8 strict classification). Forest plot of adjusted odds ratios (OR) or incidence rate ratios (IRR for positive node count) with 95% confidence intervals (CI) for HT(+) versus HT(−) across six tumor outcomes. The vertical dashed line at OR/IRR = 1.0 denotes no effect; values to the left indicate a protective association in HT(+) patients. Green markers denote statistically significant outcomes (P < 0.05); the gray marker denotes the non-significant Ki-67 outcome. *Positive node count is modeled by negative binomial regression with log(nodal yield) as offset. All primary models adjusted for age, sex, pathological diameter, and multifocality; BRAF V600E was excluded as a covariate (treated as mediator).

The principal multivariable-adjusted effects of HT across all tumor-aggressiveness outcomes are summarized in **Figure 4**. HT was independently associated with a less aggressive PTC phenotype across five domains — BRAF V600E mutation, ETE severity (ordinal), gross ETE, high CLNM ratio, and yield-adjusted positive node count — with no detectable difference in Ki-67 ≥5%.

## 4 Discussion

### 4.1 Principal findings

In this Chinese single-center cohort of 266 consecutive surgical PTC patients, concurrent HT was independently associated with a less aggressive PTC phenotype (18) across multiple biological levels: a 53% reduction in the adjusted odds of BRAF V600E mutation, a 59% reduction in the adjusted odds of ETE severity, a 79% reduction in the adjusted odds of gross ETE (T3b/T4), a 53% reduction in the adjusted odds of a high CLNM ratio, and a 44% reduction in the yield-adjusted positive node count. In contrast, HT was not associated with the proportion of patients with any CLNM, nor with the Ki-67 ≥5% rate, indicating that the protective influence of HT operates through invasion and metastatic depth rather than through tumor cell proliferation per se.

### 4.2 Consistency with the existing literature

Our results are highly concordant with the most recent meta-analytic evidence. The 2023 systematic review by Janicki et al. (8) pooled nine studies of 6,395 patients and reported an OR of 0.45 (95% CI 0.35–0.58) for BRAF V600E mutation in PTC-HT versus conventional PTC, nearly identical to our adjusted OR of 0.47. The 2025 propensity-score-matched analysis of 6,963 surgical cases by Ma et al. (10) similarly identified HT as a protective factor against lymph node metastasis, capsular invasion, and BRAF V600E mutation. The 2025 integrated meta-analysis of papillary thyroid microcarcinoma by Zhong et al. (11) found that HT reduced the central lymph node metastasis risk by approximately 25% in PTMC, broadly consistent with our finding that HT reduces nodal metastatic burden (although not the binary presence of CLNM).

Our study extends this literature in three methodologically important ways. First, we modeled ETE under strict AJCC 8 criteria (12, 13) as an ordinal outcome with three tiers, revealing a graded protective effect that would be obscured by dichotomization. Second, we applied a causal-inference framework that excludes BRAF V600E from primary multivariable models (treating it as a mediator on the HT→aggressiveness pathway), which yields larger effect estimates and clearer biological interpretation than studies that adjust for BRAF as a confounder. Third, we addressed the nodal-yield confounding concern with a negative binomial regression with log(yield) offset, demonstrating that HT reduces the rate of positive nodes per node harvested — a more biologically meaningful measure than CLNM ratio alone.

### 4.3 Putative immunological mechanisms

Several immunological mechanisms have been proposed in the literature (19) that may hypothetically account for the less aggressive phenotype observed in HT(+) PTC, although none were directly measured in the present study. Hypothetically, the dense lymphocytic infiltrate in HT may foster an antitumor immune environment via CD8+ tumor-infiltrating lymphocytes (20), IL-2-mediated MHC class I upregulation enhancing tumor antigen presentation (21), and modulation of the PD-1/PD-L1 axis (22). Regulatory T cell (FoxP3+) dynamics may also play a role (23). These remain speculative explanations that warrant direct immunohistochemical and molecular validation in future studies of our cohort.

### 4.4 Ki-67 limitations in conventional PTC

Several limitations of using Ki-67 in conventional PTC warrant discussion. First, classical PTC typically shows a low proliferative index — in our cohort, the median Ki-67 was approximately 5% with the majority of cases (n = 137, 53.3%) reported as ‘<5%’ in the original pathology reports, reflecting the limited dynamic range available to detect group differences and likely explaining the null Ki-67 finding (high Ki-67 ≥5%: 26.9% vs. 27.4%, P = 1.000). Second, intratumoral heterogeneity of Ki-67 expression and inter-observer variability in ‘hot spot’ selection are well-recognized challenges that limit reproducibility. Third, unlike in aggressive thyroid variants (poorly differentiated or anaplastic carcinoma) where Ki-67 has established prognostic value, its discriminative role in conventional PTC — particularly in microcarcinomas — remains controversial. Therefore, the absence of a Ki-67 difference between HT(+) and HT(−) groups should be interpreted in this methodological context rather than as definitive evidence against a proliferative effect of HT.

### 4.5 Methodological considerations and the AJCC 8 reclassification

ETE was classified under strict AJCC 8th-edition criteria. Pure thyroid capsule invasion was classified as no ETE and distinguished from microscopic ETE and gross ETE, avoiding inflated ETE rates and yielding estimates consistent with published cohorts. Importantly, the protective associations of HT with lower ETE severity, BRAF V600E mutation, and central lymph node metastasis remained robust under this strict classification and were strengthened in primary models excluding BRAF V600E as a mediator (avoiding overadjustment bias) (16, 24). The uniform application of prophylactic central neck dissection at our institution also reduced selection bias associated with selective CND based on clinical or radiographic suspicion.

### 4.6 Clinical implications and future directions

Our findings suggest that HT status, particularly when combined with BRAF V600E status, may stratify PTC patients into biologically distinct subgroups. However, the current dataset — single-center, retrospective, lacking long-term recurrence and survival data — does not permit translation of these biological observations into clinical management changes. Prospective validation with recurrence and survival endpoints in larger, multi-center cohorts (25) is the essential next step before any modification of surgical or adjuvant therapy algorithms could be justified.

### 4.7 Limitations

Several limitations should be acknowledged. First, this is a single-center retrospective study with a modest sample size, limiting generalizability and statistical power for some subgroup analyses. Second, HT was diagnosed on the basis of postoperative histopathology rather than preoperative serological/sonographic criteria. While this maximizes diagnostic specificity, it limits preoperative clinical applicability of our findings. Third, PTC histological variants were not consistently documented as a structured pathology field in our institutional reporting. A minority of cases (n = 15, 5.6%) had non-classic variants noted in the free-text narrative (12 follicular variant, 2 diffuse sclerosing, 1 tall cell); the remainder (n = 251, 94.4%) were presumed classic variant. As tall-cell and diffuse sclerosing variants have distinct biological behavior, the lack of systematic variant classification is a limitation. However, a sensitivity analysis restricted to the n = 251 presumed-classic subset yielded results consistent with the primary analysis (HT on gross ETE: adjusted OR = 0.15, 95% CI 0.03–0.72, P = 0.018; HT on BRAF V600E: adjusted OR = 0.50, 95% CI 0.26–0.97, P = 0.039; HT on ETE severity ordinal: adjusted OR = 0.39, 95% CI 0.18–0.88, P = 0.022), confirming that the protective association of HT is not driven by variant composition. Fourth, the TPOAb dose-response analyses are exploratory given the very small subgroup sizes. The modest Spearman correlations (r between −0.10 and −0.17) reflect weak associations whose biological meaningfulness requires confirmation in independent larger cohorts. Fifth, although our institutional ETE re-review confirmed 100% concordance with the original pathology reports, an independent re-review by an external endocrine pathologist was not performed. Finally, we lacked long-term clinical follow-up to validate the biological observations against recurrence or survival endpoints.

## 5 Conclusions

In this Chinese single-center cohort, Hashimoto’s thyroiditis was independently associated with a less aggressive PTC phenotype across multiple biological levels — reduced BRAF V600E mutation rate, reduced ETE severity under strict AJCC 8 criteria, and reduced nodal metastatic burden even after adjusting for higher nodal yield — after applying a causal-inference-informed analytic strategy. The proportional-odds assumption was supported, and findings were robust to the exclusion of non-classic histological variants. These observations strengthen the biological hypothesis that HT modulates PTC behavior through immunological pathways but require prospective multi-center validation with recurrence and survival endpoints before clinical management implications can be drawn.

## Data Availability

The raw data supporting the conclusions of this article will be made available by the authors, without undue reservation.
